# Senescence in the aging process

**DOI:** 10.12688/f1000research.10903.1

**Published:** 2017-07-25

**Authors:** Richard GA Faragher, Anne McArdle, Alison Willows, Elizabeth L. Ostler

**Affiliations:** 1 Stress, Ageing and Disease Research Group, School of Pharmacy & Biomolecular Sciences, University of Brighton, Brighton, East Sussex, UK; 2 Institute of Ageing and Chronic Disease, University of Liverpool, Liverpool, UK

**Keywords:** senescent, fibroblast, telomere, aging, lifespan

## Abstract

The accumulation of ‘senescent’ cells has long been proposed to act as an ageing mechanism. These cells display a radically altered transcriptome and degenerative phenotype compared with their growing counterparts. Tremendous progress has been made in recent years both in understanding the molecular mechanisms controlling entry into the senescent state and in the direct demonstration that senescent cells act as causal agents of mammalian ageing. The challenges now are to gain a better understanding of how the senescent cell phenotype varies between different individuals and tissues, discover how senescence predisposes to organismal frailty, and develop mechanisms by which the deleterious effects of senescent cells can be ameliorated.

## What is cell senescence?

Historically, ‘senescent’ cells are the living, but permanently non-dividing, forms of cells that are normally capable of replication within mammalian tissues. Thus, it is possible to have both growing and senescent forms of fibroblasts, keratinocytes, astrocytes, myoblasts, endothelial cells, or vascular smooth muscle cells but not mature neurones, skeletal muscle fibres, or lens fibre cells. Describing these latter cells as ‘senescent’ is usually, but not always, an unfortunate homonym that indicates that the writer is referring to material taken from an aged donor (q.v.).

The senescent state is distinct from necrosis, apoptosis, and the multiple overlapping molecular states of transient growth arrest collectively called quiescence ^1^, although linkages to these appear to be mediated through the mechanistic target of rapamycin (mTOR) axis ^2^. In biological systems where the two processes can be readily separated, principally keratinocytes, senescence can also be shown to be distinct from terminal differentiation ^3^. Senescence limits the capacity for expansion of clones of cells and thus prevents them from accumulating or expanding procarcinogenic mutations, thus acting as an *in vivo* barrier to carcinogenesis. It is thereby effectively a process-level form of the evolutionary mechanism of action termed ‘antagonistic pleiotropy’. Perhaps unsurprisingly, evidence is gradually accumulating that the short-term presence of senescent cells is beneficial in a range of normal physiological processes, including wound healing and placental development (reviewed in 4).

## How does cell senescence work?

Senescence was famously first reported by Hayflick and Moorhead as a phenomenon limiting the capacity of populations of normal human lung fibroblasts to continue to expand *in vitro* ^5^. Cell senescence has been overwhelmingly studied by using fibroblasts ever since, and this is perhaps an example of experimental tractability trouncing physiological relevance. These classic systems provided most of the contextual data and assays for senescence that were subsequently deployed in other cell types. This is perhaps best illustrated by the methods used to detect senescent cells. Early studies simply used lack of growth under conditions that would normally allow mitosis to occur. By the early 1970s and through the 1980s, two different major techniques were usually employed. The first, following largely from the work of Cristofalo, used label exclusion as the primary measure of the senescent state. This involved labelling fibroblast populations for periods significantly in excess of the cell cycle time (typically 72 hours for fibroblasts with a cell cycle time of about 24 hours) with ^3^H thymidine or later bromodeoxyuridine. A lack of label incorporation definitively indicated a senescent cell ^6^. In parallel, Hayflick, Smith, Whitney, and their associates used the isolation of individual cells and the measurement of their clonal lifespans to probe the senescent state and yielded impressive data concerning the nature of the molecular machinery ^7, 8^. This non-invasive technique for studying interclonal variation in replicative capacity could fairly be said to have reached its zenith in the work of Shall and colleagues on human and rodent mortal cell populations ^9^.

Problematically, these simple techniques did not typically allow researchers to distinguish between senescent and quiescent cells in intact tissue (Wolfe was able to combine them in studies on senescence *in vivo* to show reduced accumulation of senescent cells with calorie restriction ^10^ in the rodent lens; however, this was a technical tour de force). This lack of a utile marker for senescent cells in tissue hampered, and to some extent continues to hamper, the routine detection, quantitation, and localisation of senescent cells *in vivo*. The development of the senescence-associated β galactosidase assay by Dimri *et al*. ^11^ thus represented a significant improvement. Based on a combination of serendipity and classic catalytic histochemistry, it relies on the fact that many senescent cell types have enlarged secondary lysosomes and thus elevated β galactosidase (in fact, variations of the assay also work using substrates for dipeptidyl peptidase-2, dipeptidyl peptidase-4, and aminopeptidase m; Faragher, unpublished observations). Because catalytic histochemical techniques were originally developed to produce quantitative data on enzyme activity in tissues (significantly predating the routine use of immunocytochemistry), the senescence-associated β galactosidase assay was readily applicable to fresh tissue sections. Interestingly, alternative techniques based on other aspects of the classic senescent fibroblast phenotype such as the auto-fluorescence associated with the accumulation of lipofuscin are being developed ^12, 13^ and clearly hold considerable promise provided that users do not expect too much (‘too much’ in this instance is shorthand for “a single, perfect, rapid, easy-to-use, definitive biomarker for senescent cells that works in each and every cell type from all species under every conceivable set of conditions”).

In serially passaged cultures of human fibroblasts, senescence primarily occurs because progressive telomere shortening triggers permanent cell cycle arrest through the tumour suppressor gene p53 and the cyclin-dependent kinase inhibitor p21 ^14^. These short telomeres arise as a result both of the classic ‘end-replication problem’ associated with the replication of linear chromosomes and of the relatively low efficiency of DNA repair at telomeres ^15^. This discovery allowed the use of telomere dysfunction-induced foci (essentially short telomeres in combination with proteins, including γH2AX and 53BP1) as markers of senescence. This technique has been used to demonstrate the presence of senescent cells in primate skin and their accumulation with age but remains technically challenging ^16, 17^.

Telomere-dependent senescence is the primary mode of entry into the senescent state *in vitro* for many human cell types, including fibroblasts, retinal pigmented epithelial cells, endothelial cells, and urothelial cells ^14, 18– 20^. However, alternative pathways exist based on the action of the cyclin-dependent kinase inhibitor p16 (a selective inhibitor of cyclin D-CDK4/6 kinase pairs) ^18^ or as a direct result of the activation of p53 independent of telomere shortening ^19^. Rodent fibroblasts *in vitro* enter senescence through a pathway akin to this latter mechanism, which requires the activity of the p19 (p14 in human) ARF tumour suppressor protein (the alternate reading frame of the p16 ^INK4^ locus) and p16 itself ^21, 22^. Thus, the detection of p16 at the message or protein level can form a useful marker for the presence of senescent cells, although, as with the majority of histochemical techniques, the use of a single marker does not allow definitive assignment of the senescent state ^23^.

This basic network can be linked to a broad series of cellular stressors. These include, but are not limited to, oncogene activation, elevated levels of reactive oxygen species (stress-induced premature senescence), stress signalling in the endoplasmic reticulum (ER stress), glyoxal-induced senescence (metabolic stress), and transforming growth factor beta-induced cellular senescence (reviewed in 4). Thus, a wide range of stimuli have the potential to give rise to senescent cells in different tissues and different individuals. A complex variety of molecular mechanisms act to maintain senescence, including secreted cytokines (reviewed in 24) and altered mitochondrial function ^25^.

At least two of the three known primary senescence pathways seen in human cells are probably present in mice *in vivo*. Ironically, the only pathway that seems definitively absent in wild-type mice (as opposed to telomerase-null animals) is the telomere length-dependent senescence mechanism regularly observed in human cells ^22, 26– 28^. Rather, this appears to be present only in large, long-lived species as an additional tumour suppression mechanism. This trend can be observed within the order *Rodentia*, in which house mice and rats have long telomeres and active telomerase whereas fibroblasts from the much larger beavers, porcupines, and capybaras have telomeres much closer to human length and lack telomerase activity ^27^.

However, this does not mean that telomeric effects cannot play causal roles in cellular senescence in laboratory rodents. Although telomerase knockout mice are essentially aphenotypic for several generations because of their long telomeres, Passos and colleagues have shown that damage at the telomere is difficult to repair, resulting in telomerase-positive cells entering a senescent state when they are dosed with gamma radiation ^29^. Telomere-associated damage foci similar to those seen *in vitro* can be observed *in vivo* as laboratory mice age, suggesting that this mechanism generates senescent cells under normal conditions.

## Cell senescence causes ageing: from wishful thinking to case closed?

From its initial discovery, it was postulated that senescence, on some level, was linked with organismal ageing. Modern forms of this hypothesis propose that senescent cells are produced gradually throughout life. These then begin to accumulate in mitotic tissues and act as causal agents of the ageing process through the disruption of tissue function. This conceptual model carries three underlying assumptions: firstly that senescent cells are present *in vivo*, secondly that they accumulate with age, and finally that an accumulation of senescent cells can have a negative impact. Each is worthy of examination.

A steady production of senescent cells is quite plausible if the kinetics whereby populations of normal cells become senescent *in vitro* are assumed to be similar *in vivo*. Many early reports evaluated findings with the mistaken underlying assumption that cell cultures become senescent because all of the cells divide synchronously for a fixed number of times and then stop. In fact, it has been known since the early 1970s that each time a cell goes through the cell cycle (i) it has a finite chance of entering the senescent state and (ii) this chance increases with each subsequent division ^6– 9, 30^. Thus, senescent cells appear early on if a cell population is required to divide. Indirect demonstrations that senescent cells occur *in vivo*, accumulate with ageing, and do so at reduced rates in organisms where ageing is slowed (for example, by dietary restriction) were occasionally published from the 1970s onwards ^10, 11, 31^.

However, they were technically difficult to perform and correspondingly hard to interpret.

The presence of senescent cells would be less of a physiological handicap if they behaved in ways indistinguishable from their growth-competent counterparts. Unfortunately, senescence triggers changes in gene expression on (roughly) the same scale as differentiation from one cell type into another ^32, 33^. A central component of this shift is the secretion of biologically active proteins (for example, growth factors, proteases, and cytokines) that have potent autocrine and paracrine activities, a process termed the senescence-associated secretory phenotype (SASP) ^34, 35^. This results in cells that overproduce a wide variety of pro-inflammatory cytokines, typically through the induction of nuclear factor kappa B (NF-κB) ^36^ and matrix-degrading proteins such as collagenase ^37^. Other radical phenotypic changes, such as calcification, have also been shown to occur in some cell types with the onset of replicative senescence ^32, 38^.

The individual components of the SASP vary from tissue to tissue and, within a given cell type, can differ depending upon the stimulus used to induce senescence (for example, in fibroblasts rendered senescent by oncogene activation compared with telomere attrition or mitochondrial dysfunction) ^39– 41^. Such studies demonstrated that senescent cells could, at least potentially, produce significant and diverse degenerative pathology.

However, the observation that something can produce pathology does not mean that it *must* produce pathology, and a historic weakness of the cell senescence literature was that the *in vivo* studies essential to testing the causal relationship between ageing and cellular senescence (induced by any mechanism) were lacking. However, the production of transgenic mouse models in which it was possible to eliminate senescent cells has finally made such tests experimentally feasible. Initial studies ^42^ demonstrated first that senescent cells appeared to play a causal role in a variety of age-associated pathologies in the *BubR1* mutant mouse and subsequently that either life-long removal of senescent cells or their clearance late in life significantly attenuated the development of such pathologies in these progeroid animals ^43^. This clearly demonstrated that senescent cells can have significant, deleterious effects *in vivo.* Interestingly, the removal of senescent cells in this system was not associated with increased lifespan (an observation that demonstrated that it is possible to achieve classic ‘compression of morbidity’ by deleting senescent cells). However, on more conventional genetic backgrounds, attenuated age-related organ deterioration was accompanied by increases in lifespan of the order of 25% ^44^. A justifiable claim can be made to consider these studies ‘landmarks’ in the field in that (i) they demonstrate a causal relationship between senescent cells and ‘ageing’ and (ii) the same mechanism can cause changes associated with ‘ageing’ as well as those associated with ‘age-related disease’. These results have unusually profound philosophical implications for a scientific paper and challenge a fundamental ontological distinction that has been drawn for almost two thousand years between ‘natural’ ageing and ‘unnatural’ disease ^45^.

The interaction between senescence and the age-related loss or functional alteration of terminally differentiated post-mitotic cell populations such as skeletal muscle fibres or neurons has been relatively little studied. Such interactions could occur in at least two distinct ways: (i) paracrine effects on neurons or muscle fibres by senescent cells from adjacent mitotic compartments (for example, muscle satellite cells, fibroblasts, endothelial cells, or adipocytes) and (ii) cell-intrinsic changes in phenotype in muscle or neurons resulting from activation of the same pathways that give senescent cells their distinctive phenotype (for example, the activation of NF-κB). Given that approximately 30 to 40% of the human body is muscle, either of these could be physiologically highly significant ^46^.

In the first case, there is ample potential for senescent cells to trigger inflammatory responses in neuromuscular tissue, which then can be compounded by muscle and neuronal cells, leading to more widespread tissue dysfunction. In the second, it is already known that DNA damage in mature neurons *in vivo* induces a p21 ^waf^-mediated elevation in interleukin-6 production, conversion of euchromatin into heterochromatin, and elevated levels of lysosomal enzyme activity (analogous to that seen in senescent fibroblasts). Such damaged neurons accumulate with advancing age *in vivo* and that accumulation can be partially rescued by dietary restriction ^47^. The researchers who reported these observations termed the neuronal phenotype a ‘senescence-like state’, which is perfectly accurate but potentially confusing for those with only a cursory interest in the field. A suitably colourless neologism (perhaps *geroversion*, *gerotype*, or *senoversion*) for such phenotypic changes occurring in cell types that are already post-mitotic would seem to be called for, as would further investigation of their effects on tissue function.

## Cell senescence: where next?

Although senescent cells play an important role in tumour suppression and some unsuspected aspects of normal physiology ^48– 50^, they are central players both in age-related mortality and in various forms of morbidity (for example, as causal agents of adverse reactions to chemotherapy). However, data on their phenotype and behaviour fall far short of what is required if therapeutic progress is to be made in a reasonable timescale. For example, systematic studies on the frequency and distribution of senescent cells in human tissue analogous to those carried out on baboon skin remain to be performed (as opposed to proxy markers such as telomere length, which have been covered in more depth ^51^). The result is a literature on *in vivo* senescence in human tissues that is currently fragmentary and frustratingly difficult to interpret. Even such an apparently straightforward question such as whether the frequency of senescent cells is elevated in the skin of patients with accelerated ageing disorders such as Werner’s syndrome remains unknown. This is not because those in the field do not recognise the utility of such work; rather, it is that studies of this type are considered—in the words of one anonymous but unsympathetic referee—“boring descriptive work”. Similar issues of problem choice have occurred in other branches of ageing research at different times (for example, the merits of studying the pathology of ageing in *Caenorhabditis elegans* relative to the identification of new mutants affecting lifespan ^52^). Thus, linkages to the fraction of senescent cells required to produce degenerative effects in any given human tissue remain speculative and largely based on *in vitro* data; a figure of about 10% of the total cell population would seem to be sufficient ^53, 54^.

Whether an enhanced emphasis on basic human studies is a useful parallel-track approach to the pioneering work now taking place in rodent models or an essential next step is a matter of perspective. Many fundamental mechanisms of ageing are conserved between species (such as the insulin–insulin-like growth factor 1 axis), but there are often important species-specific differences. Those inclined to stress the cross-species similarities will be inclined to deprioritise human studies and vice versa.

Regardless of the species of origin, the extent of variation in the phenotype of senescent cells derived from the same tissue in different individuals is not well characterised. Whilst there are clearly commonalities to aspects of the senescent phenotype such as the SASP ^34, 35, 55^, it would be surprising if important intra-individual variation did not exist within the general population as well as ‘outliers’ (for example, centenarians and those with accelerated ageing diseases such as Werner’s syndrome ^56^). Although data on differential SASP profiles in response to a senescence stimulus are beginning to enter the literature, they remain fragmentary concerning the senescent cell phenotype in different tissues.

Despite these gaps, progress is being made towards the development of ‘senolytic’ drugs that can destroy senescent cells—with the goal of duplicating the effects of the transgenic mouse models first in normal animals and eventually in human patients. Initial results seem promising; for example, the BCl2 inhibitor navitoclax was shown to be senolytic in some types of senescent cells (for example, rodent fibroblasts and human endothelia) but not others (for example, human primary preadipocytes) ^57^. Recently, navitoclax was shown to provide therapeutic benefit in rodent models of atherosclerosis, mimicking the effects of senescent cell deletion models and reducing the size, number, and overall burden of atherosclerotic plaques ^58^.

Dual treatment with the senolytic compounds dasatinib and quercetin mimics the results seen in transgenic mouse senescence clearance models and improves both vasomotor function and vascular stiffness in aged and atherosclerotic mice, although the mechanistic spectrum of action of these compounds is broad and caution needs to be applied in ascribing the improvements in health status seen in senolysis alone ^59^. Quercetin also inhibits matrix metalloproteinase activity, another canonical senescence marker ^60^. This demonstrates the potential of an alternative route to dealing with the degenerative effects of senescent cells, blocking their effects rather than killing them, although improved pharmacological profiles are required before such treatments can progress further. The polyphenolic resveratrol also inhibits matrix-degrading enzymes and shows significant anti-inflammatory effects ^60– 63^. Though apparently not senolytic, resveratrol blocks both the SASP in fibroblasts and the pro-calcificatory phenotype in vascular smooth muscle cells ^61, 63, 64^. In addition, it has a range of beneficial effects on post-mitotic cell types, improving muscle mass, strength, and fatigue in rodent models ^65– 67^ and showing beneficial effects in a range of neuronal models ^68^.

Some of resveratrol’s beneficial effects appear to occur through the suppression of NF-κB activation ^62^, suggesting that blockade of this pathway may be of general benefit in treating senescence. The potential utility of selective NF-κB inhibition was recently demonstrated in *XPF-ERCC1* mutant mice, which show a wide range of progeroid features. In this model, either genetic depletion of one allele of the p65 subunit of NF-κB or treatment with a peptide inhibitor of the NF-κB activator IKK (8K-NBD, consisting of the NEMO-binding domain, fused to an 8K protein transduction domain) significantly delayed the onset of symptoms ^69^. Such studies could be usefully combined with other models of progeroid pathology, such as *BubR1*. Drugs already in clinical use, such as rapamycin and metformin, also modulate the SASP, suggesting that at least some of their beneficial effects are mediated via this route ^70, 71^.

Many aspects of the biology of senescent cells and the relationship of that biology to the aged phenotype remain to be uncovered. For example, RNA splicing factor expression alters profoundly during the ageing of both humans and rodents. This has the potential to compromise the ability of an aged tissue to produce a full spectrum of proteins in response to a physiological challenge (such as cold or heat stress). Changes to alternative splicing are also associated with replicative senescence *in vitro*, suggesting that this shift in the intact organism either results from the accumulation of senescent cells or is a parallel process affecting both senescent cells and those that have been called on to cycle repeatedly ^72^. We have extensive data showing that resveralogues reverse these changes in splicing factor expression *in vitro* and rescue multiple features of senescence independently of cell cycle traverse, SIRT1 activation, or SASP modulation. Understanding the mechanism of action of these compounds in greater detail will allow the development of small-molecule anti-degenerative treatments and provide another novel means of improving healthspan, the central goal of modern geroscience.
